# Intranasal booster induces durable mucosal immunity against SARS-CoV-2 in mice

**DOI:** 10.1038/s41598-025-06880-3

**Published:** 2025-07-07

**Authors:** Reshma Koolaparambil Mukesh, Tom Hill, Franziska Kaiser, Jessica Prado-Smith, Jonathan E. Schulz, Shane Gallogly, Lisa Herbold, Kaitlyn Bauer, Brian J. Smith, Lara Myers, Aaron B. Carmody, Carl Shaia, Vincent J. Munster, Neeltje van Doremalen

**Affiliations:** 1 Laboratory of Virology, National Institute of Allergy and Infectious Diseases, National Institutes of Health, 903 S 4th street, Hamilton, MT USA; 2 Integrated Data Science Section, Research Technology Branch, National Institute of Allergy and Infectious Diseases, National Institutes of Health, Bethesda, MD USA; 3 Rocky Mountain Veterinary Branch, National Institute of Allergy and Infectious Diseases, National Institutes of Health, Hamilton, MT USA; 4 Research Technologies Branch, National Institute of Allergy and Infectious Diseases, National Institutes of Health, Hamilton, MT USA

**Keywords:** Infectious diseases, Mucosal immunology

## Abstract

Vaccines against coronavirus disease 2019 (COVID-19), caused by severe acute respiratory syndrome coronavirus 2 (SARS-CoV-2), are highly effective in preventing severe disease but are less consistent in protecting against infection and transmission. Developing vaccines with enhanced immunogenicity that can provide protection in both the upper and lower respiratory tract (URT and LRT) is crucial. Mucosal immunization induces immunity at the site of initial infection, the respiratory tract, thereby preventing or mitigating infection. Here, we compared immune responses elicited by intramuscular mRNA vaccination alone with those elicited by intramuscular mRNA vaccination followed by intranasal administration of ChAdOx1 nCoV-19 vaccine in mice. Although both vaccination strategies induced strong systemic immunity, robust humoral and cellular mucosal immune responses, including spike-specific IgA and tissue-resident T cells, were only detected upon mucosal vaccination. Compared to unvaccinated animals, mucosal vaccination resulted in migration of T cells and macrophages into the nasal turbinates, as well as migration and proliferation of B and T cells in the nasal-associated lymphoid tissue. While both vaccination regimens provided protection across the entire respiratory tract at 2 weeks post-vaccination, at 12 weeks post-vaccination, only the mice that received a mucosal vaccination remained protected in the URT. Gene-expression profiling of the respiratory tract at 2 days post-infection revealed distinct clustering between groups. Enrichment of immune signaling pathways, including B and T cells receptor pathways, was significantly higher in intranasally vaccinated animals. Together, our study demonstrates that mucosal vaccination provides durable protection against SARS-CoV-2 than intramuscular vaccination alone.

**Supplementary Information:**

The online version contains supplementary material available at 10.1038/s41598-025-06880-3.

## Introduction

The SARS-CoV-2 pandemic has claimed more than 7 million lives^1^. Since December 2020, multiple COVID-19 vaccines have been authorized under emergency use to prevent COVID-19 disease^2^ and have been shown to reduce disease burden, hospitalization, and death^3^. These vaccines target the spike (S) protein, the receptor binding protein of SARS-CoV-2. Approved platforms include mRNA, adenovirus-vectored, and adjuvanted protein nanoparticle vaccines^4–6^. Three widely used first generation COVID-19 vaccines—BNT162b2/Comirnaty (Pfizer-BioNTech), mRNA-1273/Spikevax (Moderna), and ChAdOx1-S/Vaxzevria (AstraZeneca-Oxford)—elicited systemic neutralizing antibody responses against the SARS-CoV-2 S protein and provided 70–90% protection against severe disease^7,8^. However, continued SARS-CoV-2 transmission in the human population has driven the emergence of new variants of concern with increased immune evasion and transmissibility, thereby reducing vaccine efficacy^8–11^.

Ideally, a COVID-19 vaccine would induce a durable immune response, preventing infection in both the upper and lower respiratory tract, thereby limiting human-to-human transmission. Because SARS-CoV-2 primarily enters the body via the respiratory tract, robust immunity at mucosal surfaces in the URT is essential to prevent infection, reduce viral shedding, and interrupt transmission chains. This localized immune response could significantly enhance public health outcomes by preventing community spread, particularly during the emergence of highly transmissible variants. Traditional COVID-19 vaccines effectively induce a robust systemic immune response; however, they fail to elicit a strong mucosal immune response^12–14^. Mucosal vaccination, by inducing localized immune responses within the respiratory mucosa, can limit virus replication at the entry site and reduce transmission^7,15–20^.

In this study, we compared the immune responses elicited by a prime-boost-boost regimen, using an intramuscular (i.m.) mRNA vaccine encoding the S protein, to those elicited by a prime-boost-boost regimen consisting of two doses of i.m. mRNA vaccination followed by an intranasal (i.n.) boost with an adenovirus-vectored vaccine (ChAdOx1 nCoV-19) in mice. Since most of the USA population has received an i.m. mRNA vaccination regime, we set out to mimic this situation, and vaccinated mice initially with an mRNA vaccine, followed up with a third i.m. versus i.n. vaccination. We evaluated humoral and cellular immune responses in the upper and lower respiratory tract at D3, D14 and D84 post-vaccination. Our findings revealed strong systemic immunity across both groups, regardless of the vaccination route. However, only i.n. vaccinated animals exhibited robust humoral and cellular mucosal immune responses. These included significantly higher S-specific IgA titers and increased numbers of tissue-resident T and B cells in respiratory tissues. Mice were challenged with SARS-CoV-2-containing aerosols at D14 and D84. Both regimens provided protection across the entire respiratory tract at D14, but only mucosally vaccinated animals retained protection in the URT at D84.

Our findings indicate that immune responses elicited in the upper respiratory tract through i.n. administration of ChAdOx1 nCoV-19 may offer more durable protection than those elicited by i.m. administration of a COVID-19 mRNA vaccine, as evidenced by detectable virus replication in the URT on D2 in the i.m. group only. The durability of mucosal immunity is particularly important in the context of waning systemic immunity observed with current vaccines^21–23^, which necessitates frequent booster doses. Mucosal vaccines, by sustaining immune responses at the site of virus entry, could reduce the need for repeated vaccinations and provide a more practical solution for long-term protection.

## Results

### Mucosal administration of ChAdOx1 nCoV-19 induces durable mucosal anti-S IgA in the URT

**Fig. 1:**
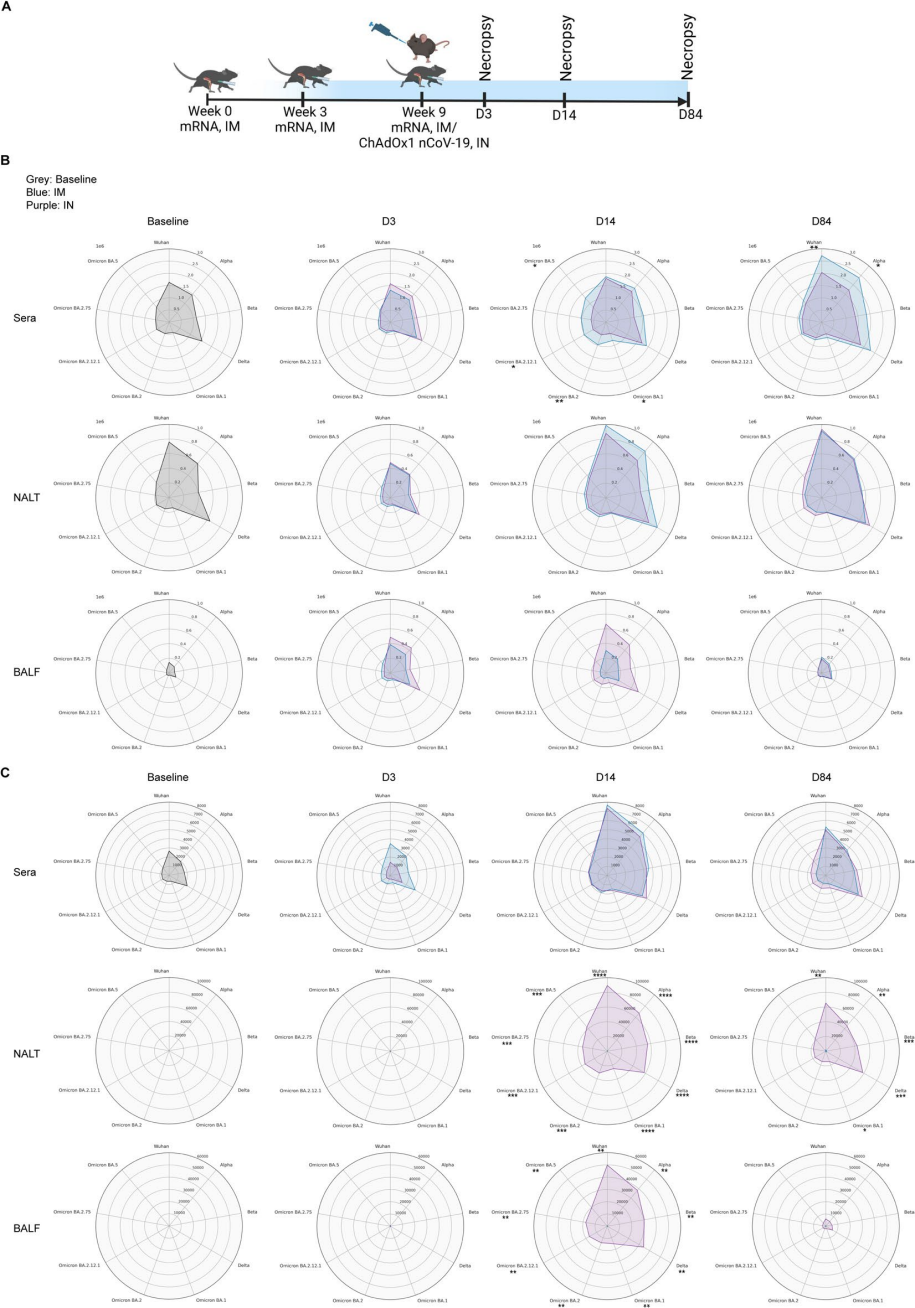
Mucosal ChAdOx1 nCoV-19 administration results in enhanced spike-specific IgA response in the upper respiratory tract. (**A**) Outline of the study. 4- to 6-week old B6 mice (male and female) were vaccinated i.m. with two doses of an mRNA vaccine (1 µg), administered 3 weeks apart. Six weeks after the second dose, the mice received either a third dose of mRNA vaccine via the i.m. route or a single dose of ChAdOx1 nCoV-19 vaccine (3.8 × 10^7^ virus particles in 25 µl PBS) via the i.n. route. Mice that received two doses of an mRNA vaccine (1 µg), administered 3 weeks apart, served as the baseline group. Sera, NALT media, and BALF were collected at baseline, D3, D14, and D84 to investigate humoral immune responses against nine different SARS-CoV-2 variants. Image created with BioRender.com. (**B,C**) Radar plots showing IgG (B) and IgA (C) levels within sera, NALT media, and BALF samples at baseline, D3, D14, and D84. The scale indicates mean fluorescence intensity values. The blue plot corresponds to the IM group, whereas the purple plot corresponds to the IN group. Statistics were performed using two-way ANOVA followed by Tukey’s multiple comparisons test. Significance between IM and IN groups are indicated as follows: * = *p*-value < 0.05; ** = *p*-value < 0.01; *** = *p*-value < 0.001; **** = *p*-value < 0.0001.

Despite the robust systemic immunity induced by i.m. administration of COVID-19 vaccines, breakthrough infections and viral transmission continue to occur. Studies have suggested that vaccination regimens capable of inducing strong mucosal immune responses, including local IgA and tissue-resident cellular immunity, may be more effective in preventing respiratory infections, including SARS-CoV-2 infection^24–27^. To investigate the ability of different vaccine routes to induce a mucosal immune response, we compared i.m. and i.n. routes of vaccination. Four- to 6-week-old C57BL/6 J (B6) male and female mice were vaccinated i.m. with two doses of an mRNA vaccine (1 µg), administered 3 weeks apart. Six weeks after the second dose, the mice received either a third dose of mRNA vaccine via i.m. administration (designated as group IM) or a single dose of ChAdOx1 nCoV-19 vaccine (3.8 × 10^7^ virus particles in 25 µl PBS) via i.n. administration (designated as group IN) (Fig. 1A). Mice that received two doses of an mRNA vaccine (1 µg), administered 3 weeks apart, served as the baseline group, while mice that received three doses of PBS were used as controls. Samples from the baseline group were collected on D0. All vaccines encoded the ancestral SARS-CoV-2 S protein.

To investigate differences in humoral responses across different tissue compartments, we collected sera, bronchoalveolar lavage fluid (BALF), and nasal-associated lymphoid tissue (NALT), a secondary lymphoid organ that runs lengthwise along the base of the nasal passage which is functionally equivalent to human tonsils^28^, from the URT at baseline, D3, D14, and D84. The collected NALTs were incubated in media for 3 days, after which the media containing secreted antibodies was harvested. Antibodies in sera represent the systemic response, those in BALF represent the lower respiratory tract response, and those in NALT media represent the URT response. Binding IgG and IgA antibody titers were measured against nine different SARS-CoV-2 variants: Wuhan (Ancestral), Alpha, Beta, Delta, and Omicron variants BA.1, BA.2, BA.2.12.1, BA.2.75, and BA.5 (Fig. 1B-C). Significant differences were determined using the two-way ANOVA followed by Tukey’s multiple comparisons test.

Since the vaccines were based on ancestral SARS-CoV-2 S protein, IgG and IgA responses across all samples were stronger against earlier variants of SARS-CoV-2 than the Omicron variants. Binding IgG titers in sera collected at baseline and on D3 were equivalent. However, binding IgG titers in sera were significantly higher in the IM group compared to the IN group for Omicron variants at D14, and for Wuhan and Alpha variants at D84. In NALT media, binding IgG titers were elevated at both D14 and D84 compared to baseline and D3. In BALF, IgG titers were highest at D3 and D14. IgG titers in NALT media and BALF were similar across both vaccine groups at all time points (Fig. 1B).

S-specific binding IgA titers in sera, NALT media, and BALF peaked at D14. In serum, there were no significant differences between the two vaccine groups at all time points tested. In stark contrast, IgA titers were significantly higher in the IN group in both NALT media and BALF. At D14, this was observed for all variants, whereas at D84, IgA titers in NALT samples remained significantly higher against Wuhan, Alpha, Beta, Delta, and Omicron BA.1, but not against later Omicron variants. IgA titers in BALF were low at D84. (Fig. 1C).

We then performed an ACE-2 competitive binding assay to quantify antibodies that block the binding of the S protein to ACE2, represented as the percentage of binding inhibition (Supplementary Fig. 1). This ACE-2 neutralization assay serves as an alternative to live virus or pseudovirus neutralization assays^29^. In line with the binding antibody profile, we observed higher inhibition of earlier SARS-CoV-2 variants compared to Omicron variants in sera. Blocking antibody titers exceeded 75% for the Wuhan, Alpha, Beta, and Delta variants at all time points including baseline. As expected, at D14, blocking antibodies against Omicron variants BA.1, BA.2, and BA.2.12.1 were significantly higher in the IM group compared to the IN group. Blocking antibody titers in NALT samples and BALF were mostly below the limit of detection (20%).

Taken together, our results underscore that mucosal administration of the ChAdOx1 nCoV-19 vaccine induces durable IgA responses in the respiratory tract, whereas i.m. administration of the mRNA vaccine alone does not. In contrast, systemic neutralizing antibody responses against earlier Omicron variants were significantly higher in the IM group compared to the IN group.

### Mucosal administration of ChAdOx1 nCoV-19 induces spike-specific tissue-resident CD8 + T cells in the URT

**Fig. 2:**
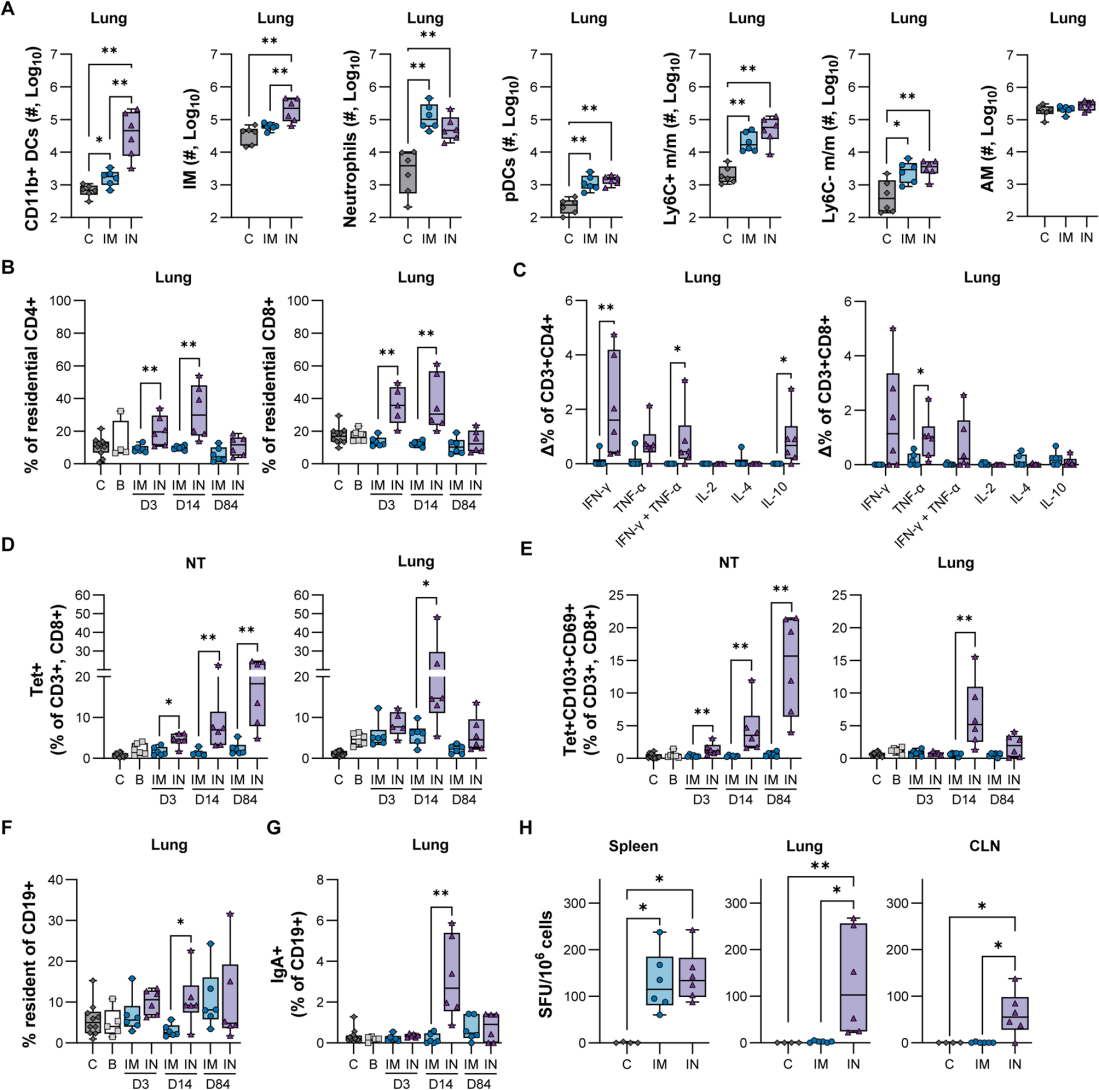
Tissue-resident spike-specific CD8 + T cells and IgA + B cells are enriched in animals that received an IN vaccination. Nasal turbinates (NT) and lung tissues were collected at baseline, D3, D14, and D84 and compared to cell populations in animals that received PBS injections. (**A**) Myeloid populations were quantified via flow cytometry in lung tissue collected at D3. (**B**) The percentage of residential CD4 + T cells (left) and CD8 + T cells (right) in lung tissue from total CD4 + or CD8 + T cells. (**C**) The percentage of residential CD4 + T cells (left) and CD8 + T cells (right) in lung tissue producing cytokines defined on the X-axis upon *ex-vivo* stimulation with SARS-CoV-2 spike peptides. (**D**) The percentage of residential spike-specific tetramer + CD8 + T cells in NT (left) and lung (right) tissue. (**E**) The percentage of residential spike-specific tetramer + CD69 + CD103 + CD8 + T cells in NT (left) and lung (right) tissue. (**F**) The percentage of residential CD19 + cells in lung tissue. (**G**) The total percentage of IgA + CD19 + cells in lung tissue. (**H**) B cell ELISpot assay showing the number of spike-specific IgA producing B cells in the spleen, lung, and CLN. A Kruskal–Wallis test followed by a Mann–Whitney test was used to determine statistical significance. * = *p*-value < 0.05; ** = *p*-value < 0.01; C = naïve animals; DC = dendritic cells; IM = interstitial macrophages; pDC = plasmacytoid dendritic cells; m/m = monocytes/macrophages; IFN- γ = interferon-γ; TNF-α = tumor necrosis factor α; IL = interleukin; Tet = tetramer; CLN = cervical lymph node.

We hypothesized that myeloid cell populations in lung tissue at D3 would be influenced by where a vaccine was administered, and which vaccine platform/route was utilized. Thus, we collected lung tissue at D3 and performed high dimensional flow cytometry. Three minutes before tissue collection, an intravenous injection of CD45 antibody (CD45 IV) was performed. This allows enough time for the antibody to circulate throughout the body and stain CD45+ cells in circulation, but not to penetrate tissues and stain resident cells. In the IN group, we observed significantly elevated numbers of CD11b+ dendritic cells (DCs, CD45 IV−, CD11b+, CD11c+, Ly6C+, F4/80+) and interstitial macrophages (IMs, CD45 IV−, CD11b+, CD11c+, Ly6C−, F4/80+, MHC Class II+) compared to the IM group as well as PBS-injected control animals. We also detected increased counts of neutrophils (CD45 IV−, CD11b+, CD11c−, Ly6G+), plasmacytoid DCs (CD45 IV−, CD11b−, CD11c+, Ly6C+, F4/80−), Ly6C+ monocytes/macrophages (CD45 IV−, CD11b+, CD11c−, Ly6G−, Ly6c+, F4/80+) and Ly6C- monocytes/macrophages (CD45 IV−, CD11b+, CD11c+, Ly6C, F4/80+, MHC Class II−) in both vaccine groups compared to controls. No differences in alveolar macrophages (CD45 IV−, CD11b−, CD11c+, Ly6C−, F4/80+) were observed between groups. Thus, we observed an influx of CD11b+ DCs and IMs into the lungs of animals that received a mucosal vaccination (Fig. 2A).

Next, we investigated the presence and functionality of CD4+ and CD8+ T cells in lung and nasal turbinate tissues. First, we determined the fraction of CD45 IV- cells within total CD4+ and CD8+ T cells in lung tissue (residential T cells, Fig. 2B). Residential T cells were similar for controls, baseline animals, and animals that received a third IM vaccination. However, lung resident CD4+ and CD8+ T cells were significantly increased at D3 and D14 in the IN group compared to the IM group, but not at D84. At D14, single cell suspensions obtained from lung tissue were stimulated with SARS-CoV-2 S peptides, and the expression of IFN-γ, TNF-α, IL-2, IL-4, and IL-10 was compared to mock-stimulated cells using flow cytometry. In resident CD4+ T cells obtained from the IN group, a significant increase in the frequency of cells producing IFN-γ and IL-10 was detected compared to cells from the IM group. Furthermore, polyfunctional CD4+ T cells, producing both IFN-γ and TNF-α, were also significantly elevated in the lungs of animals from the IN group. In contrast, only the production of TNF-α by resident CD8+ T cells was significantly elevated in the IN group compared to the IM group (Fig. 2C).

We next assessed the immune phenotype of the T cells in lung and nasal turbinate tissues collected at baseline, D3, D14, and D84 by probing tissue-resident memory (T_RM_) T-cell-specific markers, including CD44, CD103, and CD69. S-specific CD8 + T cells were sorted further using major histocompatibility complex (MHC) class I tetramer S_539-546_ (VNFNFNGL). Compared to the IM group, nasal turbinates of animals from the IN group contained a significantly increased frequency of Tet + CD8 + T cells (CD45 IV-, CD3 + , CD8 + , CD44 + , Tet +) at all three time points. Importantly, up to 21.5% of Tet + CD8 + T cells displayed co-expression of CD103 and CD69, T_RM_ signature markers, and co-expression was found to be highest at D84. In contrast, in lung tissue of animals from the IN group the frequency of Tet + CD8 + T cells (CD45 IV−, CD3 + , CD8 + , CD44 + , CD62L−, Tet +) was highest at D14, and not significantly different from those detected in animals from the IM group at D84. Accordingly, co-expression of CD103 and CD69 was highest on D14 but was reduced to below 5% at D84 (Fig. 2D,E).

T cell responses were also investigated in spleen, cervical lymph nodes (CLN, the draining lymph nodes of the URT), and inguinal lymph nodes (ILN). In CLN, Tet + CD8 + T cells numbers were relatively low at all time points throughout all groups except from the IN group at D14, and surface co-expression of CD69 and CD103 was significantly higher at this time point. Tet + CD8 + T cells in ILN were highest at D3 in the IM group, and expression of CD69 and CD103 was minimal. In spleen, the number of Tet + CD8 + T cells was significantly higher in the IM group at D3, increased to similar levels in both vaccine groups at D14, and then reduced to undetectable levels in both vaccine groups at D84. The median percentage of Tet + CD8 T cells in spleen at D14 hovered around 20% for both groups, slightly higher than that observed in lung tissue of the IN vaccinated group at this time point (14.7%). Expression of CD103 and CD69 was low, but slightly increased in the IM group compared to the IN group at D3 (Supplementary Fig. 2A).

Single cells suspensions obtained from spleen and CLN at D14 were then stimulated with SARS-CoV-2 S peptides, and release of IFN-γ or IL-2 was detected via ELISpot. In spleen, IFN-γ and IL-2 production was not significantly different between vaccine groups, and the number of cells producing IFN-γ was approximately 10–30 × higher than the number of cells producing IL-2. In CLN, only IFN-γ producing cells obtained from the IN group were above background levels (Supplementary Fig. 2B,C).

Resident B cell populations in lung tissue (CD45 + , CD19 + , CD45 IV−) were increased at D14 in the IN group compared to the IM group (Fig. 2F). Additionally, a higher percentage of these cells also expressed IgA on their surface (Fig. 2G). No differences in IgA + CD19 + CD45 IV− cells were seen in the nasal turbinates (Supplementary Fig. 2D).

The specificity and functionality of B cells was investigated in single cell suspensions of spleen, lung, and CLN tissue using a B cell ELISpot, in which the plate was coated with S protein (Ancestral variant), and IgA secretion was assessed using a 5-h incubation protocol. The number of IgA-producing plasma cells was significantly higher in the lung and CLN of the IN group compared to the IM group but was similar in the spleen (Fig. 2H).

### Mucosal vaccine administration enhances immune cell expansion and proliferation in the URT

Since an increase in immune cell populations in nasal turbinate and lung tissues was observed in the IN group via flow cytometry, we aimed to investigate whether mucosal vaccination induced similar changes in the NALT. To assess this, we primed 4- to 6-week-old B6 mice with i.m. mRNA vaccination. Three weeks later, animals were boosted with a single dose of i.n. ChAdOx1 nCoV-19 vaccine. At 3-, 7-, 14-, and 28-days post vaccination (DPV), entire heads were collected. Coronal sections at approximately the level of the first molar were prepared to visualize the NALT, and sections from vaccinated animals were compared to those from unvaccinated animals (Supplementary Fig. 3A,B). Sections were stained for CD3 (T cells), PAX5 (B cells), IBA1 (macrophages), S protein, or Ki67 (proliferation).

**Fig. 3:**
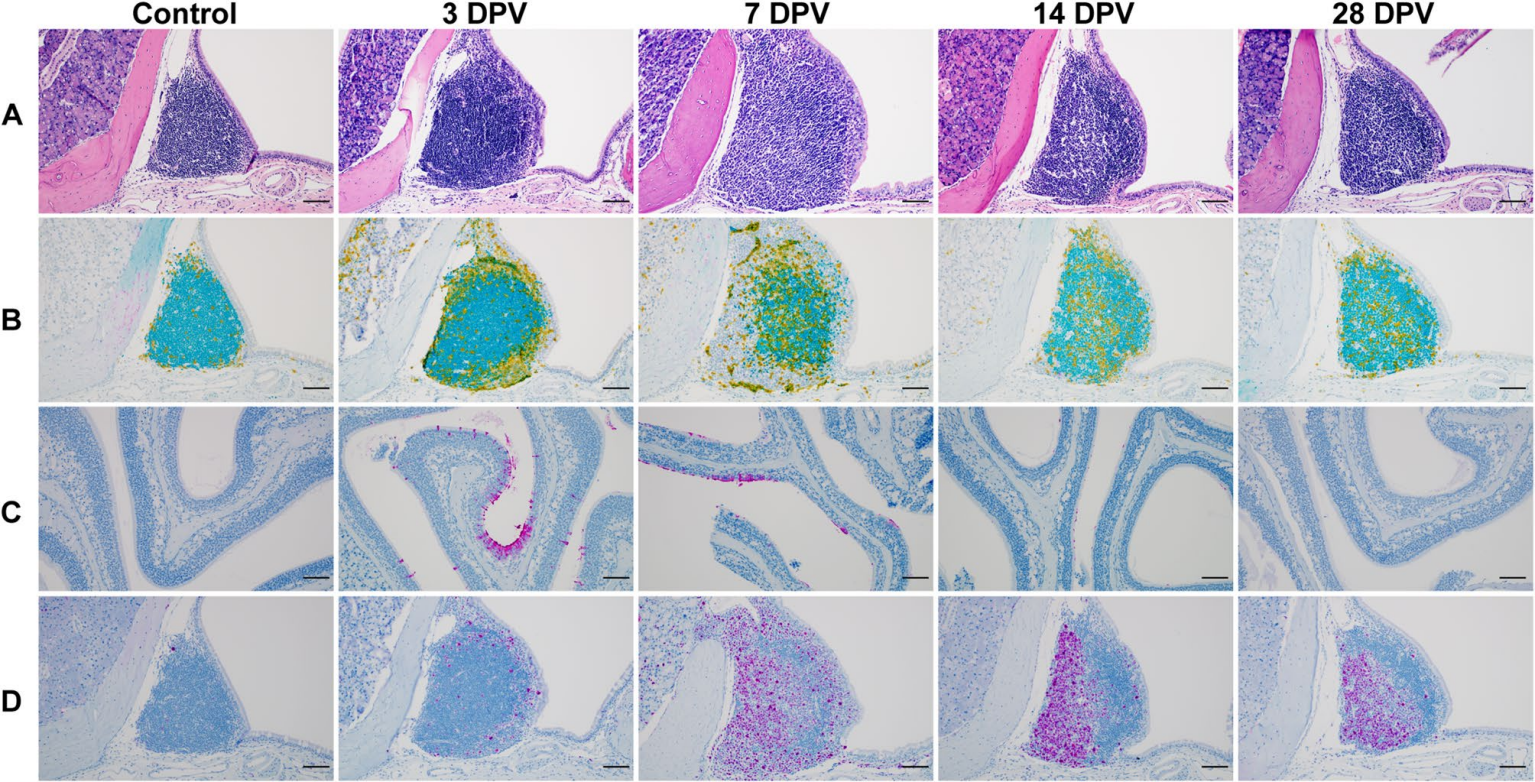
Increased size, accompanied with increased T and B cell numbers and Ki67 staining in NALT tissue of i.n. vaccinated mice. (**A**) H&E staining of NALT tissues. (**B**) IHC staining of NALT tissues with CD3 (yellow), PAX5 (teal), and SARS-CoV-2 S protein (purple). (**C**) IHC staining of nasal turbinate tissues with S protein (purple). (**D**) IHC staining of NALT tissues with Ki67 (purple). Images are representative of n = 4 mice per group. Magnification, × 200; scale bars, 100 µm.

Compared to unvaccinated controls, mucosal vaccination resulted in an increase in NALT size, with the largest size observed at 7 DPV, followed by a gradual decrease at 14 and 28 DPV (Fig. 3A, Supplementary Fig. 3C). Triplex immunostaining was performed to identify the localization of T cells, B cells, and any S protein which may be present in the vaccinated animals. Increased T and B cell staining was observed in the NALT tissues of animals that received a mucosal vaccination. In control animals, T cells were primarily localized at the periphery of the NALT. At 3 DPV, a robust increase in T cell number was observed, still predominantly located around the periphery. By 7 DPV, T cells had infiltrated the center of the NALT, where they were surrounded by B cells, and this distribution persisted at 14 and 28 DPV. Despite triplex staining showing no S protein presence in NALT, individual IHC revealed immunoreactivity in the olfactory epithelial mucosa of three out of four samples at 3 DPV and one out of three samples at 7 DPV, with no staining at 14 and 28 DPV (Fig. 3B-C). Ki67 staining showed gradual increases in cell proliferation from 3 to 28 DPV, with the highest levels at 14 DPV (Fig. 3D).

Robust T cell infiltration into the lamina propria of the nasal turbinates and sinuses was visible at 3 DPV and peaked at 7 DPV. Intriguingly, T cell staining showed aggregate formation at 14 DPV which persisted through 28 DPV (Supplementary Fig. 4A). Macrophage staining indicated increased antigen-presenting cells in the NALT starting at 7 DPV. Macrophage numbers in the nasal turbinates peaked at 7 DPV and were significantly reduced at 28 DPV (Supplementary Fig. 4B).

### Intranasally administered ChAdOx1 nCoV-19 vaccine provides long-term protection against SARS-CoV-2 infection in the URT

We demonstrate a clear distinction in mucosal immunological outcomes between the two vaccine regimens investigated in this study. To evaluate whether these differing immunological outcomes translate into differences in protection against infection, we assessed vaccine efficacy in K18-hACE2 transgenic mice. These mice express the human ACE2 gene under the control of the human epithelial cell cytokeratin 18 promoter, which is critical because SARS-CoV-2 utilizes ACE2 for cellular entry.

**Fig. 4:**
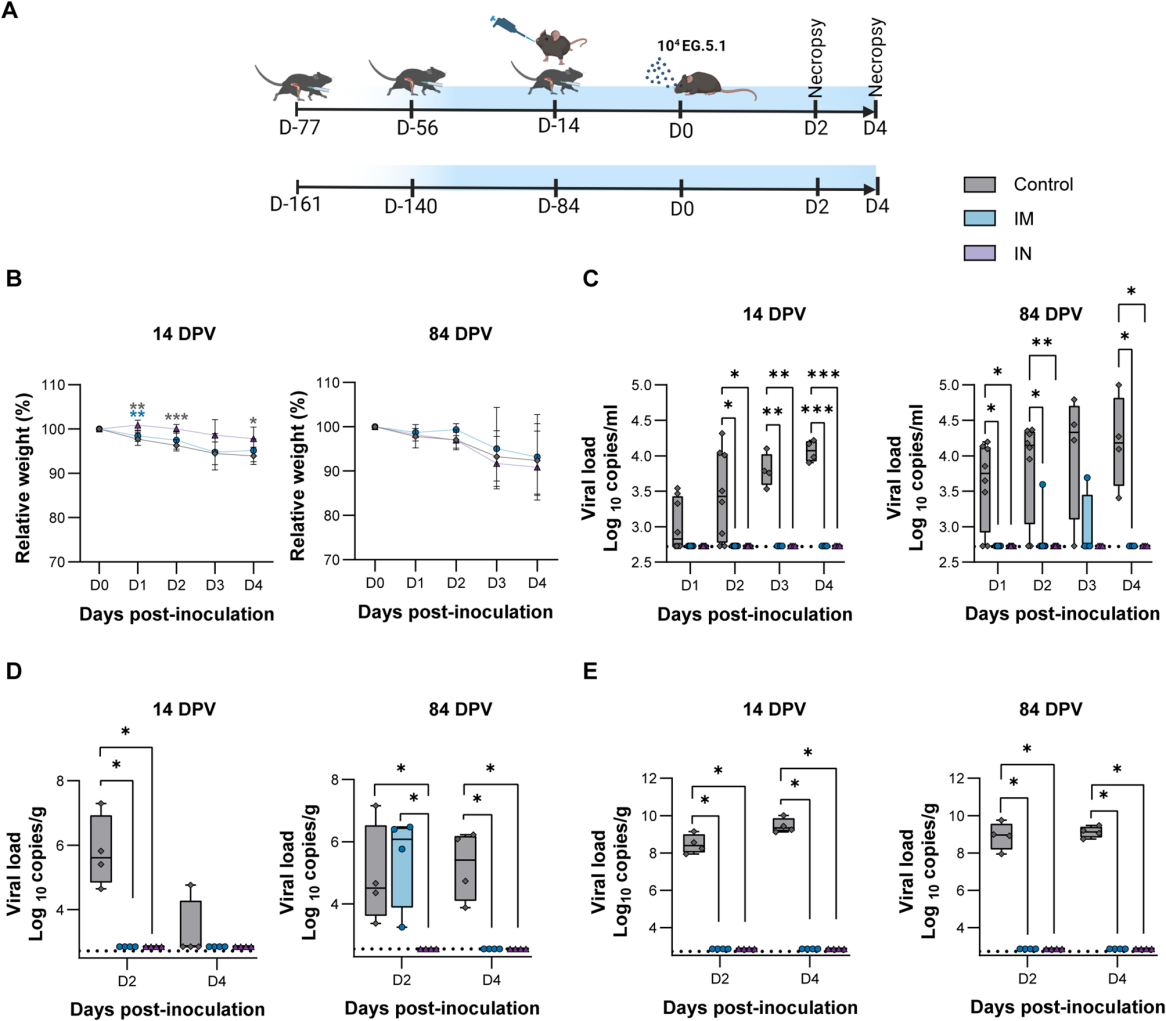
Intranasal ChAdOx1 nCoV-19 administration protects the upper respiratory tract up to 12 weeks post vaccination. (**A**) Experimental schedule of the study. Two doses of mRNA vaccination i.m. were followed by either one dose of mRNA vaccine i.m. or one dose of ChAdOx1 nCoV-19 vaccine i.n.. Two weeks (schedule on top) and 12 weeks (schedule on bottom) post-third vaccination, animals were exposed to Omicron EG.5.1 variant via aerosol. Four mice per group were euthanized at D2 and D4. Animals that received PBS served as the control group. Image created with BioRender.com. (**B**) Relative weights of the animals from D0 to D4. Significance indicated in blue and grey asterisks represents comparisons between IN vs. IM and IN vs. control, respectively **C.** Viral sgRNA present in oral swabs obtained on D1 to D4. (**D**) Viral load in NT at D2 and 4. (**E**) Viral load in lung tissues at D2 and 4. Significance was calculated using two-way ANOVA mixed-effects model followed by Tukey’s multiple comparisons test for B and C, and unpaired t-test followed by Mann–Whitney U test for figures D & E. Dotted lines indicate the limit of detection.* = *p*-value < 0.05; ** = *p*-value < 0.01; *** = *p*-value < 0.001.

Due to the unavailability of an mRNA vaccine encoding the ancestral SARS-CoV-2 S protein, this part of the study was performed using an mRNA vaccine that encodes the S protein of the XBB.1.5 subvariant of Omicron (SpikeVax, Moderna). Mice were vaccinated using the same regimen as outlined previously (Fig. 4A). Eight animals were assigned to each group, with unvaccinated animals (administered PBS via the i.m. route) serving as the control group. At 14 and 84 DPV, all animals were exposed to aerosols containing approximately 3 × 10^4^ tissue culture infectious dose 50% (TCID_50_) of Omicron EG.5.1 variant, which is antigenically closely related to XBB.1.5^30^. Mice were euthanized at D2 and D4 post exposure, with four animals per group per time point.

A significant difference in relative body weight between the IN and IM groups was observed on Day 1 in the 14 DPV group. In contrast, no differences in relative weight were observed between study groups exposed to virus at 84 DPV (Fig. 4B). High viral loads were detected in oral swabs obtained from control animals, but viral RNA was absent from swabs obtained from vaccinated animals challenged at 14 DPV. In the animals exposed at 84 DPV, viral RNA was detected in swabs from two animals in the IM group but remained undetectable in the IN group (Fig. 4C**)**.

Nasal turbinate and lung tissues collected at D2 and D4 revealed high viral load in control animals. In contrast, vaccinated animals exhibited full protection in the lower respiratory tract, regardless of regimen or time since vaccination. Nasal turbinates were also protected in both vaccine groups at 14 DPV. However, 84 DPV, viral RNA levels in the nasal turbinates of animals in the IM group were comparable to those of control animals at D2. Conversely, animals in the IN group maintained full protection. At D4, viral RNA was undetectable in the nasal turbinates of both vaccine groups, whereas it was high in the nasal turbinates of control animals (Fig. 4D-E).

Thus, regardless of vaccine regimen, all vaccinated animals were equivalently protected from infection at 14 DPV, as indicated by minimal weight loss and lack of viral RNA in the respiratory tract. In contrast, at 84 DPV, protection of the URT at D2 was observed only in animals in the IN group.

### Differentially expressed genes between tissues collected from the IN and IM groups

**Fig. 5:**
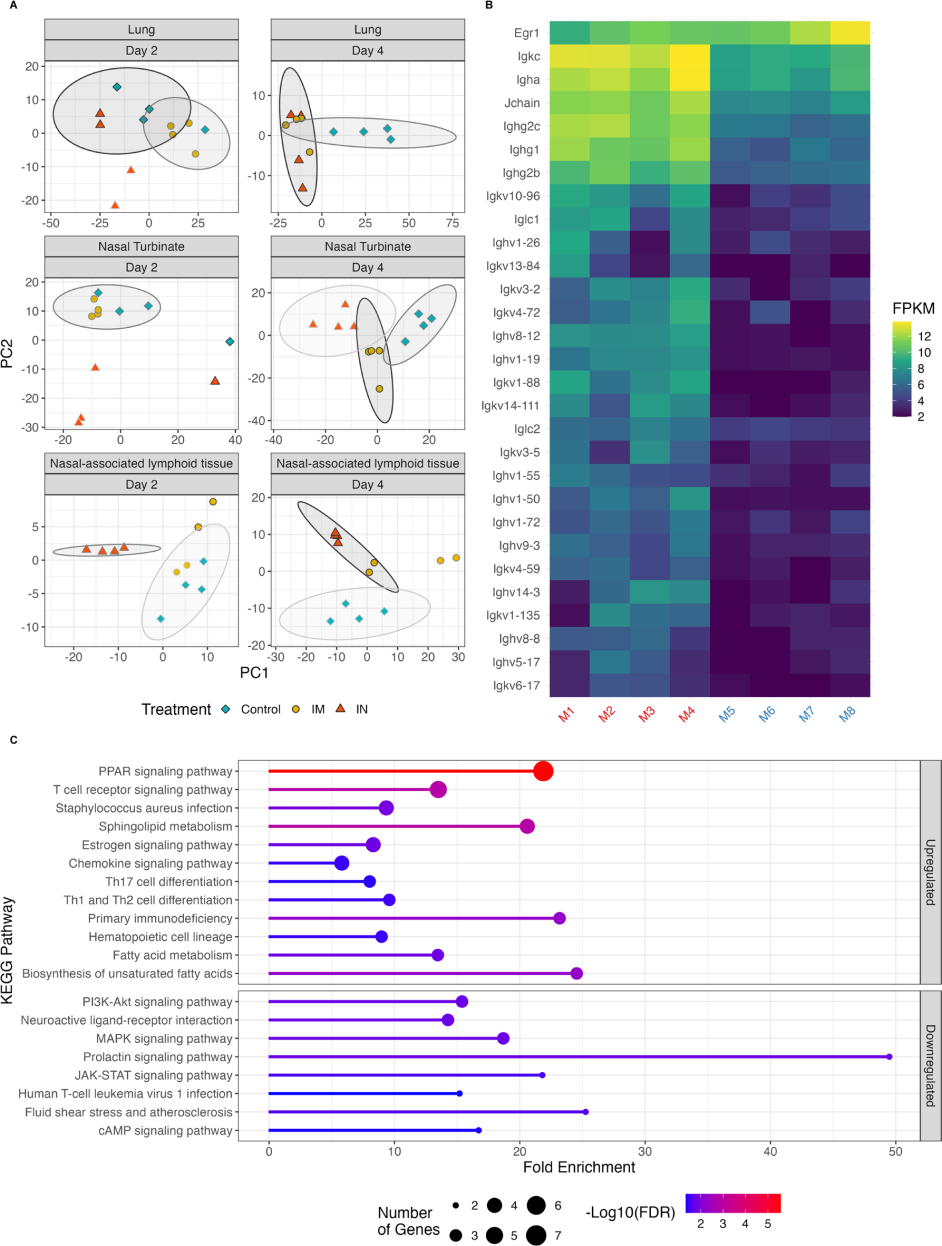
Intranasal ChAdOx1 nCoV-19 administration results in upregulation of adaptive immune responses. (**A**) Principal component analysis of samples using differentially expressed genes between vaccine groups, separated by the tissue sequenced and the day of collection post-exposure. Sample points are shaped based on vaccine type (PBS, IM, or IN). Samples were clustered based on expression similarity using the k-means clustering method, assuming three clusters. Some clusters are not encircled due to the size and dimension of the cluster. (**B**) Heatmap of expression (shown as fragments per kilobase of exon per million reads, FPKM) of B-cell receptor signaling pathway genes that are differentially expressed between IN and IM samples, collected on D2 from NT. Sample names are colored based on vaccine type, with IN (left, red) and IM (right, blue). (**C**) KEGG pathway enrichments among differentially expressed genes in the comparison between IN and IM samples, collected on D2 from NT. Pathways are separated by enrichment in upregulated or downregulated genes. Pathway color indicates the degree of significance (following FDR multiple testing correction), while the X-axis shows the fold enrichment of significant genes within that pathway compared to expectation.

Using bulk RNA-sequencing, we identified genes that were differentially expressed between vaccine groups at each timepoint post-virus challenge in nasal turbinates, NALT, and lung tissue. Numerous genes showed differential expression between the groups across timepoints (Supplementary Fig. 5). However, when clustering samples using principal component analysis (PCA) based on these differentially expressed genes, we found limited evidence that these genes fully captured group differences at each timepoint. Notably, distinct clustering by treatment was observed only in nasal turbinate samples collected at D4 (Fig. 5A). In other cases, control samples either separated from the vaccine groups (lung tissue and NALT at D4), or IN group samples clustered separately, whereas IM and control samples clustered together (nasal turbinates and NALT at D2).

Given the observed differences in virus replication in the nasal turbinates at D2, we focused our comparisons between the two vaccine groups on this time point and tissue. Among the differentially expressed genes, we found significant enrichment of B and T cell receptor signaling pathways, with a majority of B cell receptor signaling genes being upregulated in the IN group compared to the IM group. These genes included those related to antibody secretion, such as immunoglobulin light and heavy chains, and the J chain (Fig. 5B). We also identified other enriched functional KEGG pathways among the significantly differentially expressed genes between the IN and IM groups. In the IN group, several immune-associated pathways were upregulated, including genes related to Th1, Th2, and Th17 cell differentiation. Additionally, we found that multiple other pathways, including the PI3K-Akt, MAPK, and JAK-STAT signaling pathways, were downregulated compared to the IM group (Fig. 5C).

Notably, we found the peroxisome proliferator-activated receptor (PPAR), which is associated with enhanced antibody production and B cell differentiation^31^ amongst other immunoregulatory functions^32^, and the T-cell receptor signaling pathways were the most significantly enriched pathways in the IN group. Additionally, we observed a significant increase in ITGAE expression, which encodes CD103, in nasal turbinates of the IN group at D2.

Thus, we demonstrate that the IN group exhibits enhanced adaptive immune responses in the nasal turbinate at D2 compared to the IM group. These enhanced responses likely account for the observed differences in viral load in the nasal turbinate at this time point.

## Discussion

Mucosal immunity contributes substantially to provide protection against respiratory pathogens; induction of local mucosal immune responses at the site of virus entry is a pivotal strategy for limiting SARS-CoV-2 infection and onward transmission^33,34^. For instance, i.n. administered ChAdOx1 nCoV-19 encoding the S protein has previously been shown to elicit strong mucosal immunity and protection compared to the i.m. route in a murine model^18,27,35^. Our findings align with these observations, showing that the mucosal route of administration for ChAdOx1 nCoV-19 induces both robust humoral and cellular immune responses, offering durable protection in the URT. Complementary studies in rhesus macaques and hamsters demonstrated reduced viral shedding following i.n. vaccination, further supporting its role in preventing virus transmission^18,19^. By contrast, i.m. administration of vaccines in humans elicits poor mucosal immune responses. Despite eliciting strong systemic immunity, i.m. administration of mRNA vaccines, such as BNT162b2, has failed to induce robust mucosal immunity^20^, as we also observed in our studies.

A coordinated CD4 + T cell, CD8 + T cell, and antibody response is associated with milder COVID-19. In this study, we demonstrate that priming with a peripheral mRNA vaccine followed by mucosal boosting with ChAdOx1 nCoV-19 is highly effective in driving robust mucosal antibody and T cell responses in the respiratory tract. Unlike i.m. mRNA vaccination alone, mucosal vaccination effectively induces SARS-CoV-2-specific IgA antibodies and T_RM_ cells in the respiratory tract. Previous studies have highlighted the importance of IgA in early SARS-CoV-2 neutralization: IgA antibodies appear earlier than IgG in serum and exhibit a greater neutralizing response than IgG^36^. Furthermore, the relative risk of Omicron breakthrough infection was significantly reduced by the presence of mucosal IgA, but not mucosal IgG^37^. This demonstrates the unique role of IgA in promoting local immunity.

Beyond the humoral immune response, robust cellular immunity in the respiratory tract was exclusively observed animals that received a mucosal vaccination. This included higher frequencies of antigen-presenting cells and T_RM_ in the respiratory tract of the IN group compared to the IM group, consistent with studies in which i.m. mRNA vaccination was shown to be insufficient in inducing SARS-CoV-2-specific T_RM_ cells within BALF or lung tissue of humans^14,38^. Likewise, compared to convalescent patients, the proportions of CD4 + T_RM_ in the lungs of individuals who received an i.m. mRNA vaccine were modest, and polyfunctional T_RM_ were undetectable^38^. Depletion of CD8 + T cells in convalescent rhesus macaques resulted in a reduction of protection against rechallenge with SARS-CoV-2, emphasizing the critical role of cellular immunity in limiting viral spread and disease severity^14,39^. In mice, depletion of memory CD8 + T cells induced by i.n. ChAd-SARS-CoV-2-S vaccination prior to XBB.1.5 challenge reduces protection against upper and lower respiratory tract infection, highlighting the critical role of mucosal vaccine-mediated immunity in providing protection^27^.

The importance of vaccine-generated or infection-induced T_RM_ cells in providing protection against respiratory viruses has been demonstrated for respiratory syncytial virus (RSV) and influenza A virus^40,41^. In humans, higher frequencies of pre-existing CD8 + T_RM_ in the airways of healthy adult volunteers resulted in reduced disease severity and viral load after an experimental RSV infection^41^. In murine models, i.n. administration of FluMist, a live-attenuated influenza vaccine, resulted in the establishment of long-term, virus-specific CD8 + T_RM_, providing heterosubtypic protection to H3N2. In contrast, vaccination with Fluzone, an inactivated influenza virus administrated via the i.m. route, elicited durable, strain-specific humoral immunity but did not result in the establishment of T_RM_ in the respiratory tract, or heterosubtypic protection^40^.

Mucosal lymphoid structures located in the URT, such as the NALT, play a critical role in limiting respiratory infections by initiating robust mucosal immune responses at the primary site of pathogen entry. We observed significant alterations in T and B cell populations within the NALT following mucosal vaccination. Previous studies have shown that i.n. immunization effectively induces antigen-specific protective immunity against respiratory viruses, activating both mucosal and systemic immune compartments^42–44^. scRNA-seq performed on NALT from animals immunized with a single-dose i.n. vaccination using lyophilized S protein revealed that NALT serves as a key inductive site following i.n. vaccination, promoting the differentiation of B and T cells into structures resembling germinal centers^44^. Our findings align with this study, as evidenced by increased immune cell proliferation, expansion within the NALT, and the spatial organization of these cells into germinal center-like structures, as suggested by the restructuring of T and B cells.

Virus challenge after vaccination showed that the mucosal SARS-CoV-2 booster vaccine provided superior and more durable protection in the URT compared to i.m. mRNA vaccination. This was associated with a stronger adaptive immune response at D2 in the nasal turbinates of animals in the IN group. Since SARS-CoV-2 infection initially occurs in the URT, protecting the URT is hypothesized to control further virus spread to the lower respiratory tract, thereby preventing severe disease, and limit virus transmission. Studies conducted in various preclinical animal models indicated that mucosal vaccination could reduce or completely prevent virus transmission^19,45–47^. Mucosal immunization of primary contact hamsters with ChAd-CoV-2-S reduced SARS-CoV-2 in the upper and lower respiratory tract and prevented subsequent transmission to vaccinated and unvaccinated hamsters, indicating the potential of mucosal vaccines in curbing virus spread^19^. Although we did not pursue virus transmission in this study, this would be a relevant follow-up study.

Based on DEG profiling, we showed increased antibody production and T cell activation in the nasal turbinates of animals that received a mucosal vaccination compared to those that only received a peripheral vaccination. Interestingly, transcriptomic analyses revealed the upregulation of the PPAR signaling pathway in mucosally vaccinated animals. The PPAR signaling pathway consists of three small molecule receptors (PPARα, PPARδ, and PPARγ), which act as ligand-activated transcriptional regulators. PPARs are involved in lipid metabolism, but expression has been found in macrophages, DCs, B cells, and T cells^48–51^. In T cells, PPARγ ligand exposure combined with antigen and antigen-presenting cells results in significant inhibition of proliferation and increased apoptosis^52^, possibly associated with the downregulation of IL-2 production^53^. In contrast, PPARγ is upregulated in activated B cells, and associated with B cell proliferation, plasma cell differentiation, and antibody production^31^. Increased PPARγ expression in bone marrow-derived DCs after vaccination with a PST adjuvant was associated with higher antibody serum levels^54^. These findings suggest a potential role for PPAR signaling in promoting mucosal immunity, warranting further study.

In this study, we investigated mucosal immunology in the upper and lower respiratory tract and discovered critical differences between these two components. While antibody titers in BALF contracted between D14 and D84, they remained relatively stable in NALT media. Furthermore, T_RM_ populations were more durable in the nasal turbinates than in lung tissue. It is well-documented that T_RM_ populations in lung tissue are less stable compared to other tissues (Reviewed in^55^). The URT has been less extensively studied, but the relative durability of T_RM_ responses, as we observe in this study, were reported after influenza infection in mice^56^. Understanding the drivers behind the maintenance of plasma cell and T_RM_ populations, as well as the role of mucosal immune responses in both the upper and lower respiratory tract, will be crucial in elucidating their contributions to protection against viral infection and subsequent vaccine design. In nonhuman primates, two doses of an i.m. ancestral S mRNA vaccine followed by an i.n. bivalent ChAd-SARS-CoV-2-Wuhan-1 and ChAd-SARS-CoV-2-Omicron BA.5- vaccine, administered 7 months later, elicited robust IgA and pulmonary T cell responses. Interestingly, whereas IgG, IgA, and T cell responses were protective in the LRT, only mucosal IgA was associated with protection in the URT^57^. Although difficult to untangle, further research is needed to elucidate the precise roles and mechanisms of local IgA and T_RM_ cells in SARS-CoV-2 immunity.

Overall, our data demonstrates that boosting with an i.n. ChAdOx1 nCoV-19 vaccine induces robust mucosal immune responses in the respiratory tract, offering durable protection. This highlights the importance of next-generation vaccines which induce robust mucosal immunity and establishing strong cellular defenses at the site of infection. While this study was conducted in a murine model, the parallels with human studies, such as limited T_RM_ cell induction by mRNA vaccines in BALF or lung tissue^40^, underscore the translational relevance. In contrast with studies in nonhuman primates^18,57^, a Phase I trial conducted to investigate the immunogenicity of an i.n. administered adenovirus-vectored COVID-19 vaccine in vaccine-naive participants and those previously vaccinated with i.m. SARS-CoV-2 vaccines demonstrated inefficient induction of both mucosal and systemic antibody responses^58^. Given the complexity of the human respiratory mucosa, which includes protective barriers such as mucus and cilia, optimizing vaccine delivery, enhancing antigen absorption, and incorporating adjuvants could significantly improve the induction of immune responses. Based on our results, incorporating mucosal vaccination as a booster in immunized individuals could complement existing systemic immunity and provide comprehensive protection against both infection and severe disease. Human clinical trials will be crucial to further delineate appropriate mucosal vaccine strategies. Finally, mucosal vaccines also offer practical advantages. Intranasal administration is less invasive, potentially increasing vaccine uptake, especially in pediatric populations or needle-phobic individuals.

We acknowledge several limitations to our study. While we investigated the ability of an i.n. booster vaccine to provide protection against SARS-CoV-2, we did not explore its efficacy in preventing transmission. Additionally, our focus was primarily on the protection conferred by the i.n. ChAdOx1 nCoV-19 booster vaccine following i.m. mRNA vaccination. Further studies are needed to compare vaccine regimens using differing routes but the same vaccine platform and the extent of cross-protection against multiple SARS-CoV-2 variants. Since the primary objective of this study was to develop a mucosal vaccination model and explore the immune responses elicited by different vaccination strategies, we chose to utilize the ancestral SARS-CoV-2 S protein, as both the vaccine and animal model were readily available at the time. It would be valuable to confirm that the immune responses observed with the ancestral SARS-CoV-2 S protein are also elicited when using antigens from variants of concern, potentially combined with initial vaccination with ancestral antigen.

Due to the unavailability of the ancestral SARS-CoV-2 S mRNA vaccine, we conducted our challenge study using a currently available mRNA vaccine encoding the XBB.1.5 S protein. The potential for enhanced protection via cross-reactive neutralizing antibodies induced by heterologous antigens warrants further investigation. Although several studies have shown that SARS-CoV-2 infection itself can induce humoral and cellular immune responses, we focused specifically on identifying immune correlates of protection conferred by vaccination. It is also important to note the anatomical differences between the URTs of humans and mice, which may impact the translatability of i.n. vaccination^59^. For example, humans lack NALT but instead have tonsils located in a different anatomical region. Furthermore, the amount of olfactory epithelium is significantly reduced in humans compared to mice, and our study observed predominant S protein expression in the olfactory epithelium of the mice after i.n. vaccination. The implications of these structural differences on vaccine efficacy are not fully understood and require further research^60^.

Several studies have investigated the benefits of intranasal boosting following intramuscular priming, with a primary focus on lung protection and humoral immune responses in the upper respiratory tract. However, studies investigating the role of local cellular immunity in mediating protection within the URT are limited. While the underlying immune induction mechanisms and the specific correlates of protection in this region remain poorly understood, our study provides new insights. Our study distinguishes itself from these studies by the experiments conducted to explore the local immune components in the URT, including the immunohistochemical analysis of NALT.

In conclusion, our findings underscore the potential of intranasal mucosal vaccines, such as ChAdOx1 nCoV-19, to complement systemic immunity by inducing robust local immune responses at the respiratory tract, offering durable protection against SARS-CoV-2 infection and transmission. This intranasal vaccination model is intended to provide a foundational understanding of the immune responses to intranasal vaccination, which could inform the development of more effective countermeasure strategies in the future. Although we did not assess protection beyond 84 days, it is possible that the decline in protection in the URT observed in the IM group compared to continued protection in the IN group would become even more apparent with extended follow-up. Our study highlights the critical need for advancing mucosal vaccine strategies to enhance immune defenses at the site of pathogen entry, ultimately contributing to improved public health outcomes globally.

## Materials and methods

### Ethics statement

All animal experiments were conducted after obtaining prior approval from the Institutional Animal Care and Use Committee of Rocky Mountain Laboratories, National Institutes of Health. Experiments were carried out in an AAALAC international accredited facility, following the guidelines and basic principles in the Guide for the Care and Use of Laboratory Animals, the Animal Welfare Act, US Department of Agriculture, and the US Public Health Service Policy on Humane Care and Use of Laboratory Animals. C57BL/6 J (B6) and B6.Cg-Tg(K18-ACE2)2Prlmn/J (K18-hACE2) mice (Jackson Laboratory strain# :034860) were used in this study. The animal room was climate-controlled with a fixed light–dark cycle of 12-h light/12-h dark. The Institutional Biosafety Committee (IBC)–approved work with infectious SARS-CoV-2 virus strains under biosafety level 3 (BSL3) conditions. All sample inactivation was performed according to IBC-approved standard operating procedures for the removal of specimens from high containment. The study is reported in accordance with ARRIVE guidelines.

### Cells, vaccines and viruses

SARS-CoV-2 EG5.1 (hCoV-19/USA/CA-Stanford-147_S01/2023) variant was used for the challenge study. Virus propagation was performed in VeroE6 cells in DMEM supplemented with 2% fetal bovine serum, 1 mM l-glutamine, penicillin (50 U/ml) and streptomycin (50 μg/ml; DMEM2). The used virus stock sequence was 100% identical to the initial deposited GenBank sequence, and no contaminants were detected. VeroE6 cells were maintained in DMEM supplemented with 10% fetal bovine serum, 1 mM l-glutamine, penicillin (50 U/ml), and streptomycin (50 μg/ml; DMEM10). Mycoplasma testing was performed at regular intervals, and no mycoplasma was detected.

This study used a chimpanzee adenoviral-vector-based vaccine that encodes the S (ChAdOx1 nCoV-19) and an S-encoding mRNA-LNP vaccine. ChAdOx1 nCoV-19 vaccine was obtained from Professor Teresa Lambe and Professor Sarah Gilbert, University of Oxford. The mRNA LNP vaccine (Helix Biotech) was obtained from Dr. Pamela Bjorkman, Caltech, or was purchased at a local pharmacy.

### Animal studies

1. *Vaccination and immune response analysis* Six animals per group of B6 mice (4 to 6 weeks old male and female) were anesthetized with isoflurane and vaccinated with two doses of 1 ug mRNA encoding the SARS-CoV-2 S protein in 100 µl via the i.m. route at 0 and 3 weeks. At week 9, animals were vaccinated either i.n. with 25 µl of ChAdOx1 nCoV-19 (3.8 × 10^7^ virus particles) or i.m. with the same mRNA encoding the SARS-CoV-2 spike protein. As a negative control, mice were injected with PBS via the i.m. route. Mice were euthanized via bilateral thoracotomy at D3, D14 and D84. Tissues including NALT, nasal turbinates, cervical lymph nodes, inguinal lymph nodes, lungs and spleens were collected to check immune responses. Sera, BALF, and media incubated with NALT samples were collected to investigate systemic immune responses. Three minutes prior to euthanasia, animals were anaesthetized with ketamine-xylazine and injected IV via retro-orbital sinus with 2 µg of labelled CD45 antibody (Biolegend, cat#103,151) to stain leucocytes, allowing us to distinguish between circulating and extra-vascular leucocytes.
2. *Vaccination and IHC on NALTs for immune cell detection* Four animals per group of B6 mice (4 to 6 weeks old male and female) were anesthetized with isoflurane and vaccinated with one dose of i.m. mRNA vaccination. Three weeks later, animals were boosted with a single dose of i.n. ChAdOx1 nCoV-19 vaccine. At 3, 7, 14, and 28 DPV, animals were anesthetized with isoflurane, euthanized via cervical dislocation, and entire heads were collected. Coronal sections at approximately the level of the first molar were prepared to visualize the NALT by IHC.
3. *Vaccination and virus challenge* Eight animals per group of K18-hACE2 mice (4 to 6 weeks old male and female) were anesthetized with isoflurane and vaccinated with mRNA encoding the S protein at 0 and 3 weeks. At 9 weeks, animals received a 3^rd^ dose of vaccine either 25 µl of ChAdOx1 nCoV-19 i.n. or 100 µl of mRNA encoding the SARS-CoV-2 S protein i.m. At 11 and 21 weeks, unanesthetized animals were challenged with SARS-CoV-2 EG5.1 variant via aerosols. Briefly, we inoculated with a virus dose of 2 × 10^7^, which was calculated to yield an inoculation dose of 1 × 10^5^ in mice at the approximate weight of 20 g. Mice were placed in a 19.5L aerosol containment chamber, which was stationed inside a BSC and exposed to the aerosolized inoculum at a rate of 6L/min for 10 min. The Aerosolizer used was the Aero3G aerosol management platform (Biaera Technologies) and used a three jet aerosolizer inside a collison to generate aerosols. Once the exposure was complete, the mice were removed from the containment chamber and transported back to their normal cages. Back titration of virus inoculum showed that at 11 weeks, animals received 2.95 × 10^4^ TCID_50_ of virus per animal, whereas at 21 weeks, animals received 5.31 × 10^4^ TCID_50_ of virus per animal. Post-infection, animals were anesthetized with isoflurane and weighed and swabbed daily (oropharyngeal). Four animals from each group were anesthetized with isoflurane and euthanized via cervical dislocation at 2- and 4-days post-challenge, upon which tissues were collected and processed for further analysis.

### NALT antibody collection

The upper palate of the mice containing the NALT was dissected and transferred into a 48-well plate containing 250 μL of complete culture medium (RPMI 1640 supplemented with 10% fetal bovine serum, 1 mM l-glutamine, penicillin (50 U/ml) and streptomycin (50 μg/ml), gentamicin (50 μg /mL), fungizone/amphotericin (2 μg/mL)). The tissue was washed in the media several times, and the plates containing the palates were incubated at 37 °C for 3 days. The culture media collected on day 3 was centrifuged at 380 × g for 10 min and stored in − 80 for bulk antibody analysis.

### Flow cytometry

Post-labelling with CD45 antibody, mice were euthanized, and tissues were harvested. Collected tissues, including nasal turbinates, cervical and inguinal lymph nodes, lung, and spleen, were processed, single-cell suspensions were prepared and allowed to pass through 70 and 100 µm filters. Briefly, the tissues were either minced using sharp scissors (lung and nasal turbinates) or gently crushed using the back of a syringe plunger (lymph nodes and spleen). Liberase (Sigma Aldrich, cat#5401127001) treatment followed with trituration using a 3 mL syringe and 18G 1 inch needle were performed on lung and nasal turbinate tissues to ensure proper digestion and cell dissociation. Ammonium-chloride-potassium (ACK) buffer (Biolegend, cat#420302) was used to lyse red blood cells from the samples. Cells were counted using trypan blue and cell counter, and the concentration was adjusted to 10^7^ cells/mL. 100–150 µl of the suspension was added per well of a 96-well plate and further immunolabelled for surface and intracellular markers. Controls included unlabeled cells and single antibody labeled cells along with appropriate fluorescence minus one (FMO). Cells were then analyzed using a BD SymphonyA5 (BD Biosciences). Flow cytometric analysis was performed using FlowJo (BD Biosciences). The antibodies used are as follows: CD3 (145-2C11), CD4 (GK1.5), CD8 (53-6.7), CD11b (M1/7015), CD11c (HL3), CD19 (1D3), CD44 (IM7), CD45 (30-F11), CD62L (MEL-14), CD69 (H1.2F3), CD103 (2E7), F4/80 (BM8), IA/IE (M5/114.15.2), IFNγ (XMG1.2), IgA (1040-07), IgD (11-26c.2a), IgM (II/41), IL-2 (JES6-5H4), IL-4 (11B11), IL10 (JES5-16E3), Ly6C (AL21), Ly6G (1A8), TNFα (MP6-XT22), Tetramer S539-546 (VNFNFNGL).

### ELISpot assay

IgA secreting B cells and IFNγ/IL-2 secreting T cells were detected using a mouse double-colour ELISpot assay kit (ImmunoSpot, OH, USA). For B cell ELISpot, plates were coated on day 1 with 80 µl/well of anti-6xhis tag antibody (Biolegend, cat#652505) at 10 µg/mL in PBS and incubated overnight at 4 °C. On day 2, plates were washed with PBS and coated with 80 µl/well of S-his (Acro Biosystems, cat# SPN-C52H9-500ug) at 10 µg/mL in PBS and incubated overnight at 4 °C. On day 3, after washing with PBS, 3 × 10^5^ cells/wells were plated on the capture antibody pre-coated plate and incubated for ~ 6 h (B cell assay) and at 37 °C.

For T cell ELISpot, plates were coated with 80 µl/well murine IFN-γ/IL-2 Capture Solution on day 1 and incubated overnight at 4 °C. On day 2, 100 µl/well Antigen/mitogen solutions were added followed by the addition of 3 × 10^5^ cells/wells in 100 µl solution. Cells containing plates were incubated for 24 h at 37 °C.

Post-incubation, IgA or IFNγ/IL-2 specific FITC and biotin conjugated detection antibody solution was added and incubated for 2 h at room temperature. After incubation, a tertiary solution containing alkaline phosphatase labelled streptavidin, and horseradish peroxidase labelled FITC was added and incubated for 1 h in the dark at room temperature. Later, substrate solutions were added, and the formation of colored (blue and red) spots was monitored. The frequency of colored spots corresponding to cytokine-producing cells was determined and analyzed by CTL ImmunoSpot reader and ImmunoSpot Software.

### Meso scale discovery multiplex assay

SARS-CoV-2 S-specific IgG and IgA antibody levels in sera, BALF, and NALT media were detected using MSD V-PLEX SARS-CoV-2 Key Variant Spike Panel 1 mouse IgG (K15655U-2) and IgA (K15657U-2) Kits. The V-PLEX SARS-CoV-2 Key Variant Spike Panel 1 contains antigens from Alpha, Beta, Delta, and several sublineages of Omicron. The dilutions were optimized according to the sample type and assay and followed the standard protocol provided in the kit. Sera were diluted to 10,000 and 5,000 times to detect anti-spike IgG and IgA, respectively; whereas BALF and NALT media were diluted to 500 and 200 times to detect anti-spike IgG and IgA, respectively.

The presence of neutralizing antibodies against SARS-CoV-2 in sera, BALF, and NALT samples was measured indirectly using the V-PLEX SARS-CoV-2 Key Variant Spike Panel 1 ACE2 Kit (K15654U-2). The ACE2 competitive binding assay quantitatively measures antibodies that block the binding of ACE2 to its cognate ligands. Sera samples were diluted 100 times and BALF and NALT samples were diluted 20 times to measure the neutralizing antibody levels against nine different antigens coated on the plate. The results obtained were shown as percent inhibition, calculated using the following equation:

$$\% {\text{inhibition}} = 1 - {\text{average}}\;{\text{sample}}\;{\text{ECL}}\;{\text{signal}}/{\text{average}}\;{\text{ECL}}\;{\text{signal}}\;{\text{of}}\;{\text{calibrator }}8\left( {{\text{diluent}}\;{\text{only}}} \right) \times 100$$

### Histology and immunohistochemistry

NALT was collected by first removing the mouse head and mandible followed by the skin of the skull and placing the remaining skull in. Collected tissue samples were perfused with 10% neutral-buffered formalin for fixation for a minimum of 7 days. Tissues were processed with a Sakura VIP-6 Tissue Tek and sectioned at 5 µm. Sectioning generally presented three similar but slightly different anatomical appearances to the NALT and surrounding palate, septum and turbinals. For continuity across histologic evaluation the sections were categorized based on the level of section across the nasal septum and were labeled as either “complete” where the nasal septum was connected to the palate (most rostral); “tapered” where the nasal septum and palate had separated and the tip of the septum was tapered (caudal to the “complete” septum sections); and “blunt” where the nasal septum becomes wide at the tip and begins to flare laterally (considered the most caudal section). Photomicrographs were obtained primarily from tapered sections and occasional blunt sections as the tapered and blunt sections often provided a more representative cross section of the NALT. NALT size measurements were accomplished by outlining the perimeter of each NALT follicle using the Olympus Standard CellSens software’s measurement tool and were recorded in square micrometers (um^2^). Immune cells, including T cells, B cells, macrophages, and dendritic cells, were stained using antibodies specific to CD3(2GV6) (cat# 790-4341, Roche Tissue Diagnostics), PAX5 (Cat# NBP2-38790, Novus Biologicals), SARS-CoV-2 Spike S1 subunit protein (Cat# 40150-R007), IBA1 (Cat# ab5076, Abcam), and Ki67 (SP6) (Cat# GTX16667, GeneTex). The tissues were then processed for immunohistochemistry using the Discovery Ultra automated stainer (Ventana Medical Systems) with a ChromoMap DAB kit (cat#760-159) for all markers except Ki67 and S protein. Roche Tissue Diagnostics Discovery purple kit (Cat# 760-229) was used to stain Ki67 and S protein.

Triplex staining was performed for T cells, B cells and S protein using CD3 (2GV6) (Cat# 790-4341), PAX5/BSAP (Cat #NBP2-38790) and anti-SARS-CoV Spike S1 subunit protein (Cat# 40150-R007). CD3, PAX5/BSAP and S protein were stained with Roche Tissue Diagnostics DISCOVERY Yellow kit (Cat# 760-239), Roche Tissue Diagnostics DISCOVERY Teal kit (Cat# 760-247) and Roche Tissue Diagnostics Discovery purple kit (Cat# 760-229), respectively.

### RNA extraction and quantitative reverse-transcription polymerase chain reaction

Sub-genomic (sg) viral RNA was detected by qRT-PCR^61^. RNA was extracted from swabs using a QiaAmp Viral RNA kit (Qiagen) according to the manufacturer’s instructions. Lung and nasal turbinates were homogenized and extracted using the RNeasy kit (Qiagen) according to the manufacturer’s instructions. Viral sgRNA specific primers were used for the detection of viral RNA. RNA (5 μl) was tested with the TaqMan™ Fast Virus One-Step Master Mix (Applied Biosystems) using QuantStudio 3 Flex Real-Time PCR System (Applied Biosystems) according to instructions of the manufacturer. Dilutions of SARS-CoV-2 standards with known genome copies were run in parallel to construct a standard curve and calculate copy numbers/mL or copy numbers/g. The detection limit for the assay was 5 copies/reaction, and samples below this limit were considered negative.

Primer sequences are provided below:

Forward primer: 5ʹ CGATCTCTTGTAGATCTGTTCTC 3ʹ.

Reverse primer: 5ʹ ATATTGCAGCAGTACGCACACA 3ʹ.

Probe: 5ʹ FAM-ACACTAGCCATCCTTACTGCGCTTCG-ZEN-IBHQ 3ʹ.

### Transcriptomics

To quantify the expression of each gene, we mapped our bulk RNA sequencing data to the MM10 genome using the RNA-seek pipeline version 1.9.0 (https://github.com/skchronicles/RNA-seek). Briefly, this pipeline assessed the quality of each sample using FastQC v0.11.9^62^, Preseq v2.0.3, Picard tools v2.17.11^63^, FastQ Screen v0.9.3^64^, Kraken2 v2.0.8^65^, QualiMap^66^, and RSeQC v2.6.4^67^. Adapters and low-quality sequences were trimmed using Cutadapt v1.18^68^. The trimmed reads were aligned against version M21 of the mm10 Mus musculus genome and annotation, using the splicing-aware aligner STAR version 2.7.6a^69^ in per-sample two pass basic mode. We then estimated gene and transcript expression levels via RSEM v1.3.3^70^.

After finding the read counts per gene and isoform, we normalized the measurements to account for differences in the number of reads per sample and to account for the differences in gene lengths. We first weighted by the number of million fragments per sample to generate a transcripts per million measure (TPM) per gene per sample, and then divided these measures by the number of thousand exon base pairs of each gene to get a measure of fragments per kilobase per million reads (FPKM).

To identify expression differences between control and treated cases, we used iDEP (http://bioinformatics.sdstate.edu/idep95/) to normalize expression and then identify genes which significantly differ in expression between treatments using the DEseq2 algorithm, after accounting for sample as a covariate.

We generated figures from these results using R.

### Statistical analysis

Statistics were performed and significance was calculated as indicated where appropriate using GraphPad Prism 10 Software. *p*-values less than 0.05 were considered significant.

## Electronic supplementary material

Below is the link to the electronic supplementary material.

Supplementary Material 1

## Data Availability

All data have been deposited on Figshare: https://figshare.com/s/ac36bfc0e4fc27ac28e7.
