# Supplementary material for: Intranasal booster induces durable mucosal immunity against SARS-CoV-2 in mice
Source: Sci Rep. 2025 Jul 7;15:24224. doi: 10.1038/s41598-025-06880-3 (PMC12234998; doi:10.1038/s41598-025-06880-3)
Supplement: Supplementary file 1 — Supplementary Material 1 [file 41598_2025_6880_MOESM1_ESM.pdf]

Intranasal booster induces durable mucosal immunity against SARS-CoV-2 in mice

Reshma Koolaparambil Mukesh[1], Tom Hill[2], Franziska Kaiser[1], Jessica Prado-Smith[3], Jonathan E. Schulz[1], Shane Gallogly[1], Lisa Herbold[3], Kaitlyn Bauer[3], Brian J Smith[3], Lara Myers[4], Aaron B. Carmody[4], Carl Shaia[3], Vincent J Munster[1], Neeltje van Doremalen[1*]

1. Laboratory of Virology, National Institute of Allergy and Infectious Diseases, National Institutes of Health, Hamilton, MT, USA
2. Integrated Data Science Section, Research Technology Branch, National Institute of Allergy and Infectious Diseases, National Institutes of Health, MD, USA
3. Rocky Mountain Veterinary Branch, National Institute of Allergy and Infectious Diseases, National Institutes of Health, Hamilton, MT, USA
4. Research Technologies Branch, National Institute of Allergy and Infectious Diseases, National Institutes of Health, Hamilton, MT, USA

*Corresponding author: Neeltje van Doremalen, 903 S 4th street, Hamilton, MT, USA.
neeltje.vandoremalen@nih.gov

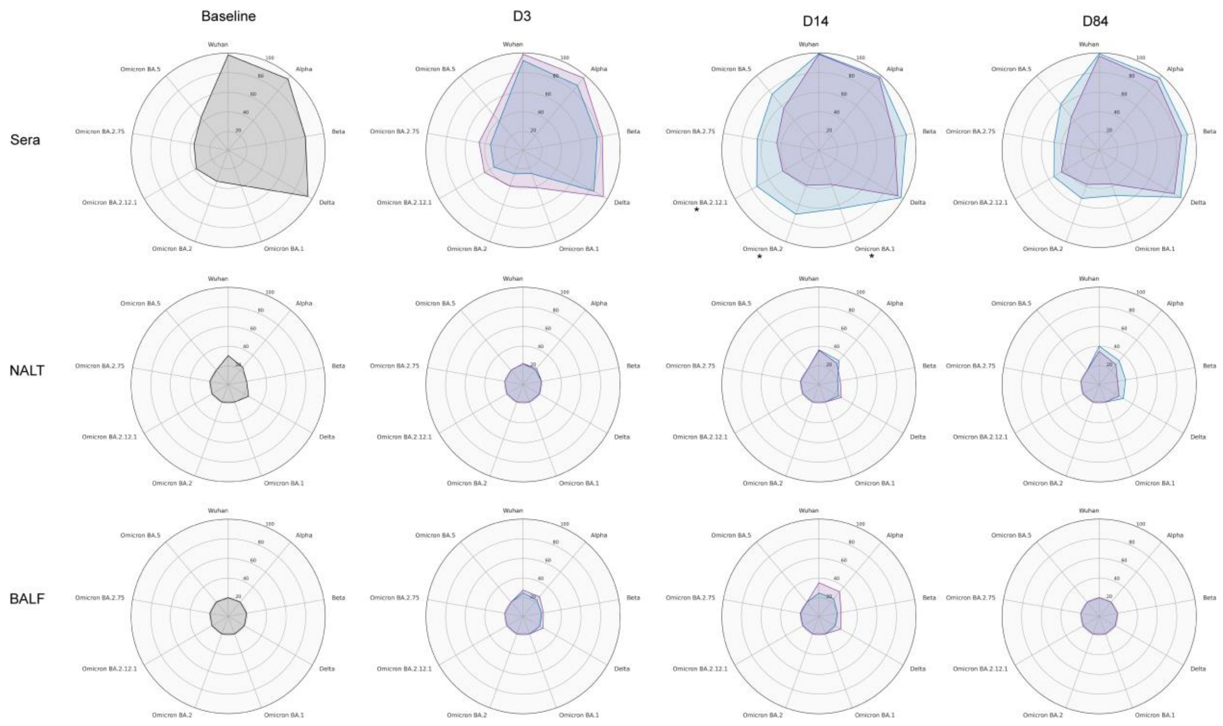

Supplementary figure 1. ACE-2 competitive binding assay. Radar plot demonstrating the percentage blocking of ACE-2 binding by antibodies present in the sera, NALT media, and BALF at baseline, D3, D14, and D84 after vaccination. Blue plot corresponds to the data from samples obtained from group IM and purple plot corresponds to the data from samples obtained from group IN. The scale indicates percentage blocking of ACE-2 binding by antibodies. The limit of detection is 20%. Statistics were performed using two-way ANOVA followed by Tukey's multiple comparisons test. * = p-value<0.05.

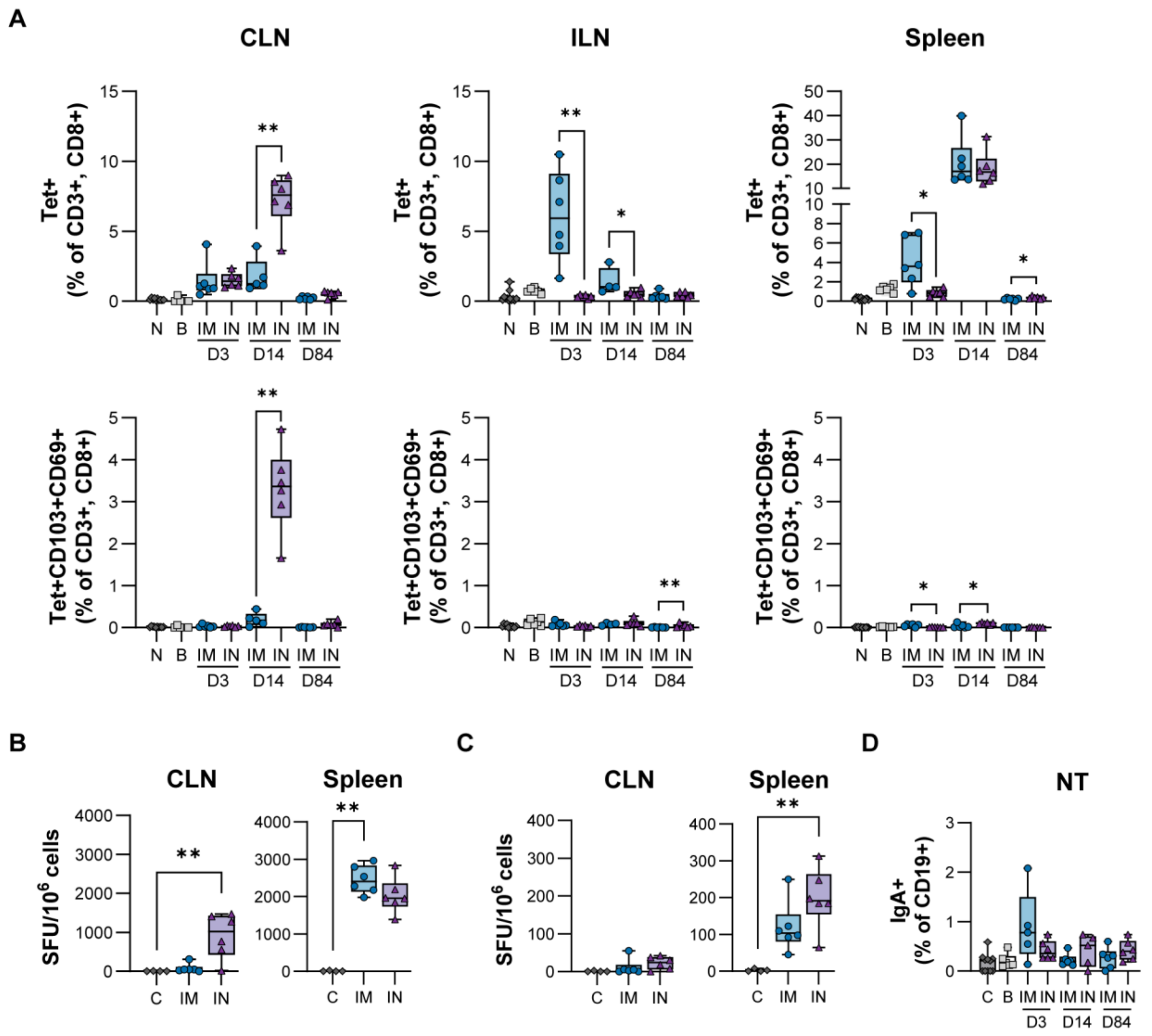

Supplementary figure 2. Tissue-resident spike-specific CD8+ T cells are enriched in CLN, but not ILN and spleen tissue, of animals that received an IN vaccination. CLN, ILN, and spleen tissues were collected at baseline, D3, D14, and D84 and compared to cell populations in animals that received PBS injections. **A.** The total number of residential spike-specific tetramer+ CD8+ T cells in tissues (top panels) and the total number of residential spike-specific tetramer+ CD69+ CD103+ CD8+ T cells (bottom panels) in tissues. **B-C.** T cell ELISpot assay showing the number of S-specific IFNγ producing T cells (B) and S-specific IL-2 producing T cells (C) in the spleen and CLN. **D.** The total percentage of IgA+ CD19+ cells in NT tissue. Significance was calculated using Kruskal–Wallis test followed by a Mann-Whitney test for A

and Kruskal–Wallis test followed by Dunn's multiple comparisons test for B and C. * = p-value<0.05; ** = p-value<0.01; C = naïve animals; Tet = tetramer.

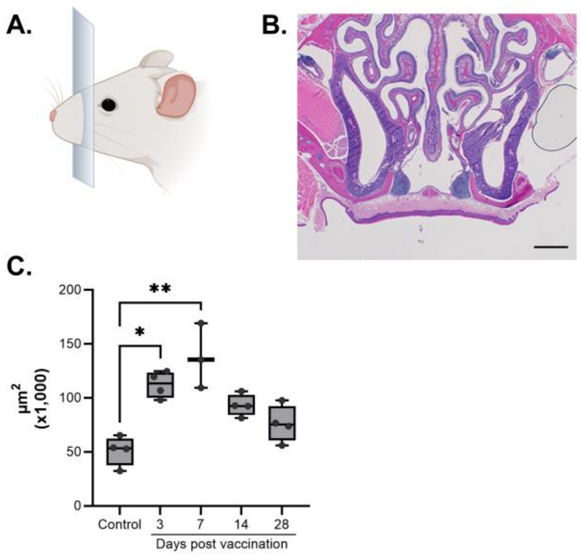

Supplementary figure 3. NALT sectioning of mice. A. Schematic representation of a coronal section of a mouse head. **B.** Example of a H&E stain of a NALT and nasal turbinates section (×20; scale bar, 1 mm). **C.** Size of NALT was measured for all animals and surface area was calculated. A Kruskal–Wallis test followed by a Mann-Whitney test was used to determine statistical significance. * = p-value<0.05; ** = p-value<0.01.

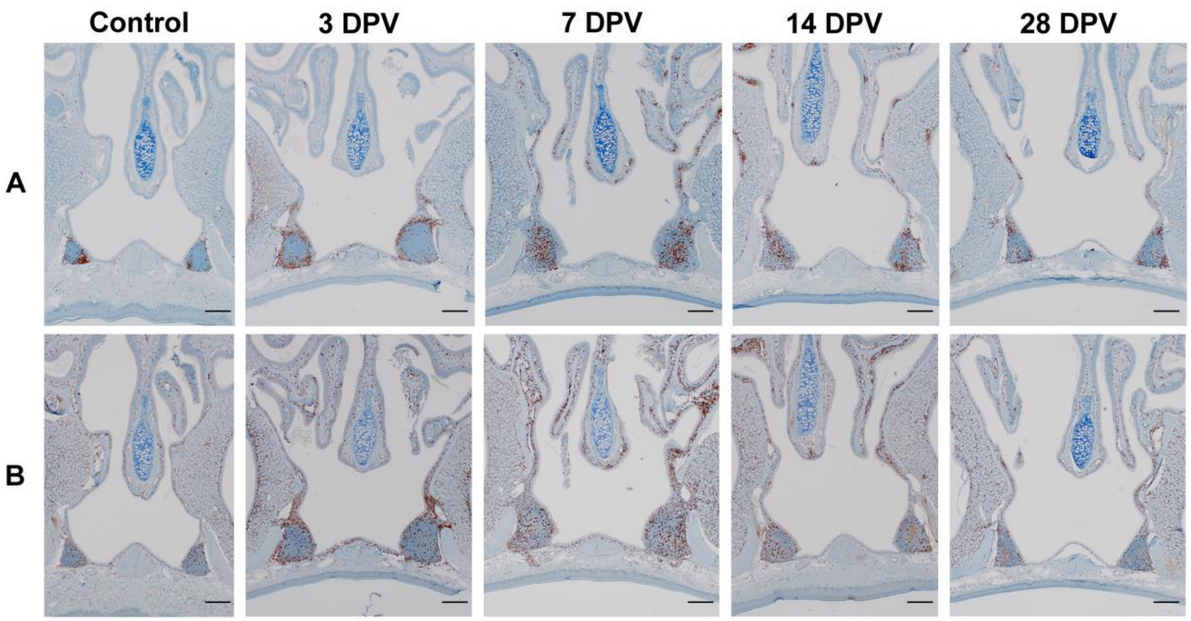

Supplementary figure 4. Migration of T cells and macrophages into nasal turbinates and NALT. A. IHC staining of nasal turbinate and NALT tissues with CD3 (brown). **B.** IHC staining of nasal turbinate and NALT tissues with IBA1 (brown). Images are representative of n = 4 mice per group. Magnification, ×40; scale bars, 500 µm.

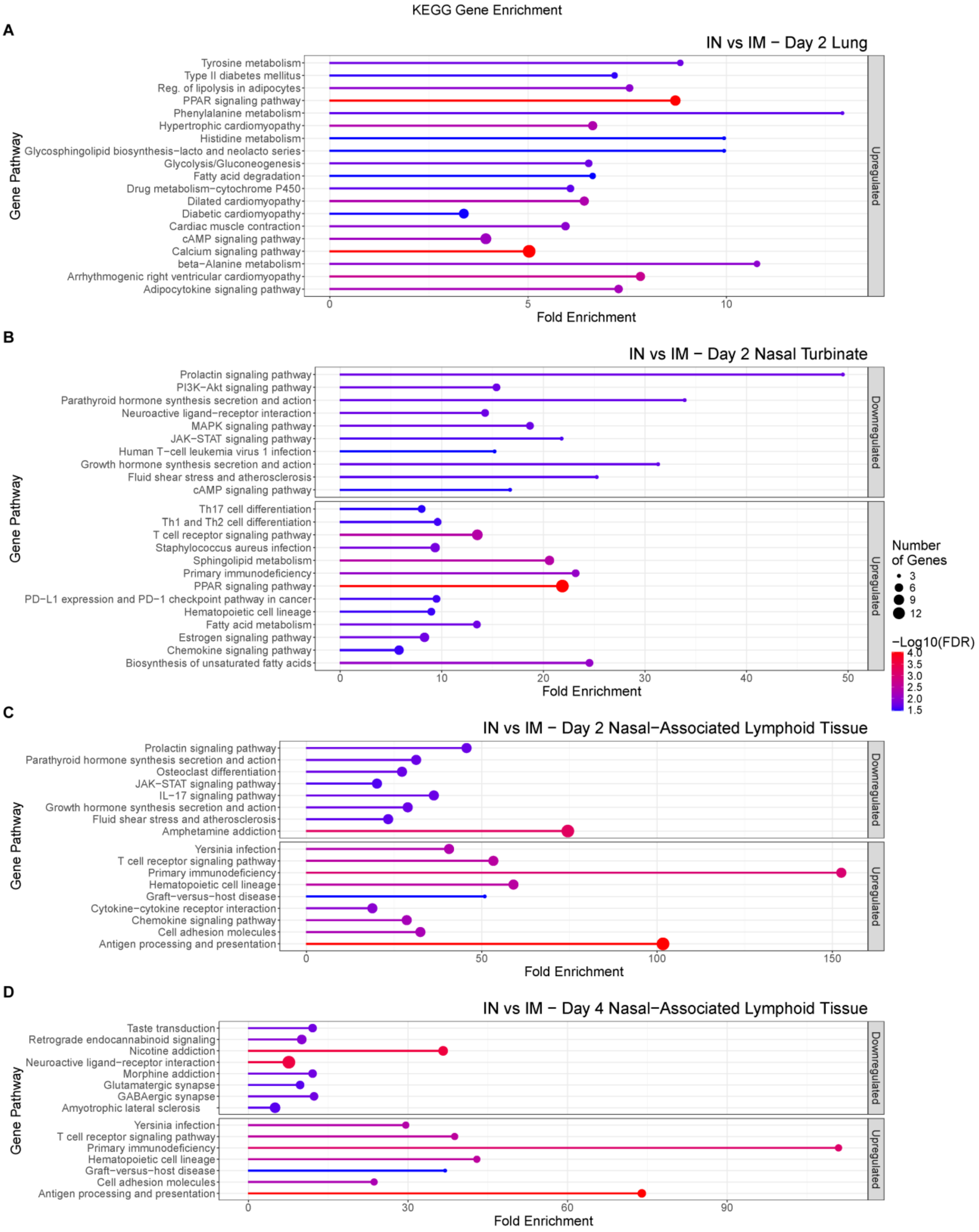

Supplementary figure 5. Gene pathway enrichment analysis using the Kyoto Encyclopedia of Genes and Genomes (KEGG) for different differential expression analyses results for IM and IN samples.

Results are separated into genes which are significantly upregulated and downregulated. The significance of each pathway (multiple-testing corrected FDR p-value) is shown in the color of the bar, from blue to red, from least to most significant. The length of each bar shows the fold-enrichment of each pathway above the expected number of significant genes when not associated. The number of significant genes in each functional category is shown by the point size. **A/B/C**. KEGG enrichment for lung samples (A), nasal turbinates (B) and NALTs (C) from day 2. **D.** KEGG enrichment for NALTs from day 4.
